# Efficacy of extended view totally extra peritoneal approach versus laparoscopic intraperitoneal on lay mesh plus for abdominal wall hernias: a single center preliminary retrospective study

**DOI:** 10.1186/s12893-023-02098-0

**Published:** 2023-07-13

**Authors:** Haisong Xu, Wenhao Huang, Yuehua Guo, Mingyue Li, Gongze Peng, Tianchong Wu

**Affiliations:** 1 The Second Clinical Medical College, Jinan University, Shenzhen, China; 2 Department of Hepatobiliary and Pancreatic Surgery, Shenzhen People’s Hospital, The Second Clinical Medical College, Jinan University, Shenzhen, 518020 Guangdong Province China; 3 The First Affiliated Hospital, Southern University of Science and Technology, Shenzhen, China

**Keywords:** Extended view totally extra peritoneal (e-TEP), Laparoscopic intraperitoneal on lay mesh (IPOM) plus, Abdominal wall hernia

## Abstract

**Background:**

Laparoscopic minimally invasive surgery has become the primary treatment for ventral hernias. The laparoscopic intraperitoneal on lay mesh (IPOM) plus approach for abdominal wall hernias is the most used procedure, while extended view totally extraperitoneal (e‑TEP) repair is a newer option. This study aimed to compare the effectiveness and complications of the 2 procedures for abdominal wall hernias repair.

**Methods:**

This was a retrospective and comparative single-center study done at The Second Clinical Medical College, Jinan University Hospital (Shenzhen People’s Hospital), Shenzhen, China. The study included patients with a 2 to 6 cm abdominal wall defect who underwent hernia repair from January 2022 to December 2022. Patients’ baseline characteristics, hernia features, operative time, blood loss, postoperative pain level, and total hospitalization expenses were extracted from the medical records and compared between patients who underwent the IPOM plus and e-TEP repair.

**Results:**

A total of 53 patients were included: 22 in the e-TEP group and 31 in IPOM plus group. Patient demographic characteristics were similar between the 2 groups. The operation time of the e-TEP groups was significantly longer than the IPOM plus (98.5 ± 10.7 min vs. 65.9 ± 7.3 min, *P* < 0.01). Postoperative pain levels (VAS; visual analog scale) (4.2 ± 0.9 vs. 6.7 ± 0.9, *P* < 0.01), analgesic requirements (Tramadol) (25.0 ± 37.0 mg vs. 72.6 ± 40.5 mg, *P* < 0.01), length of hospital stay (1.2 ± 0.5days vs. 2.2 ± 0.6days, *P* < 0.01), and total hospitalization expenses (19695.9 ± 1221.7CNY vs. 35286.2 ± 1196.6CNY, *P* < 0.01) were significantly lower in the e-TEP group. The mean intraoperative blood loss was similar between the 2 groups. No postoperative complications were observed in either group.

**Conclusion:**

The e-TEP approach for abdominal wall hernias appears to be better than IPOM plus with respect to postoperative pain levels(VAS: 4.2 ± 0.9 vs. 6.7 ± 0.9, P < 0.01), analgesic requirements(25.0 ± 37.0 mg vs. 72.6 ± 40.5 mg, P < 0.01), length of hospital stay(1.2 ± 0.5days vs. 2.2 ± 0.6days, P < 0.01), and hospitalization costs (19695.9 ± 1221.7CNY vs. 35286.2 ± 1196.6CNY, P < 0.01).

**Supplementary Information:**

The online version contains supplementary material available at 10.1186/s12893-023-02098-0.

## Introduction

A hernia is defined as when a tissue or organs leaves their usual location through an aponeurotic defect to another body cavity, and hernia treatment has evolved over many years [1]. Ventral hernias are very common; a report in 2012 from the Cochrane Collaboration indicated the number of ventral hernia repair surgeries in Europe was about 400,000 per year and about 300,000 in the United States, and the number is expected to increase by more than 10,000 per year globally [2]. These data suggest that the burden of ventral hernias is slowly increasing [3]. Minimally invasive operations have become increasingly common since the introduction of laparoscopic surgery [4]. Patients who undergo a laparoscopic procedure tend to recover faster, with an earlier resumption of activities, less pain, and fewer wound complications than those who receive an open procedure [5–7]. Laparoscopy was first described for hernia treatment and proven to be safe and effective in 1992 [8]. Since then, laparoscopic minimally invasive hernia repair has become the procedure of choice [9].

From laparoscopic intraperitoneal on lay mesh (IPOM) to extend view totally extraperitoneal (e-TEP), Minimally invasive surgery for ventral hernia repair has evolved rapidly in the last decade [10]. The most common method is laparoscopic intraperitoneal on lay mesh (IPOM) plus, in which the defect is closed by placing a mesh against the abdominal wall inside peritoneal cavity. IPOM plus is a relatively simple procedure, and a popular treatment for abdominal wall hernias [11]. Compared to the classical laparotomy surgical approach, the advantages of IPOM plus are apparent. However, shortcomings of IPOM plus include erosion of the mesh, adhesion of the mesh to the bowel, acute and chronic pain, and rarely an enterocutaneous fistula [12].

In 2012, extend view totally extraperitoneal (e-TEP) laparoscopic inguinal hernia repair was introduced [13]. The technique was adopted for ventral hernia repair in 2018 [14].The e-TEP approach does not require placing a mesh in the abdominal cavity, it placed the mesh in the anterior peritoneal space thus avoiding many complications associated with the IPOM plus approach. Notably, the mesh material requirements for e-TEP are much lower than for IPOM plus, which can reduce the surgery cost [15]. The e-TEP technology has become popular in these years, while the outcomes of e-TEP are encouraging, definitive studies are lacking [16–18].

Few clinical studies have compared the outcomes of e-TEP and IPOM plus for the treatment of abdominal wall hernias. Thus, the purpose of this study was to compare the short-term effects of the two surgical procedures about surgical data and outcomes, complications, and hospital costs for hernias repair, initially verify the safety and reliability of e-TEP. The result of this study can be used as reference materials for clinical decision-making.

## Materials and methods

### Data collection

The medical records of patients who underwent IPOM plus or e-TEP for the repair of an abdominal wall hernia from January 2022 to December 2022 were retrospectively reviewed. In order to reduce heterogeneity, only patients with midline primary, umbilical, or incisional hernias with a defect size of 2–6 cm were included in this study. Patients who received emergency surgery, a recurrent hernia, an incarcerated hernia, skin infections, and those with serious underlying diseases were excluded. Before the surgery, we will provide patients with surgical options including traditional open surgery, IPOM, transabdominal preperitoneal hernioplasty (TAPP) and e-TEP, fully inform them of the indications, risks and technical maturity of the different surgical options. The patient’s questions about the procedure were carefully answered, and the final surgical plan was chosen by the patient. The study was approved by the hospital Ethics Committee, and the requirement of informed consent was waived due to the retrospective nature of the study.

**Fig. 1:**
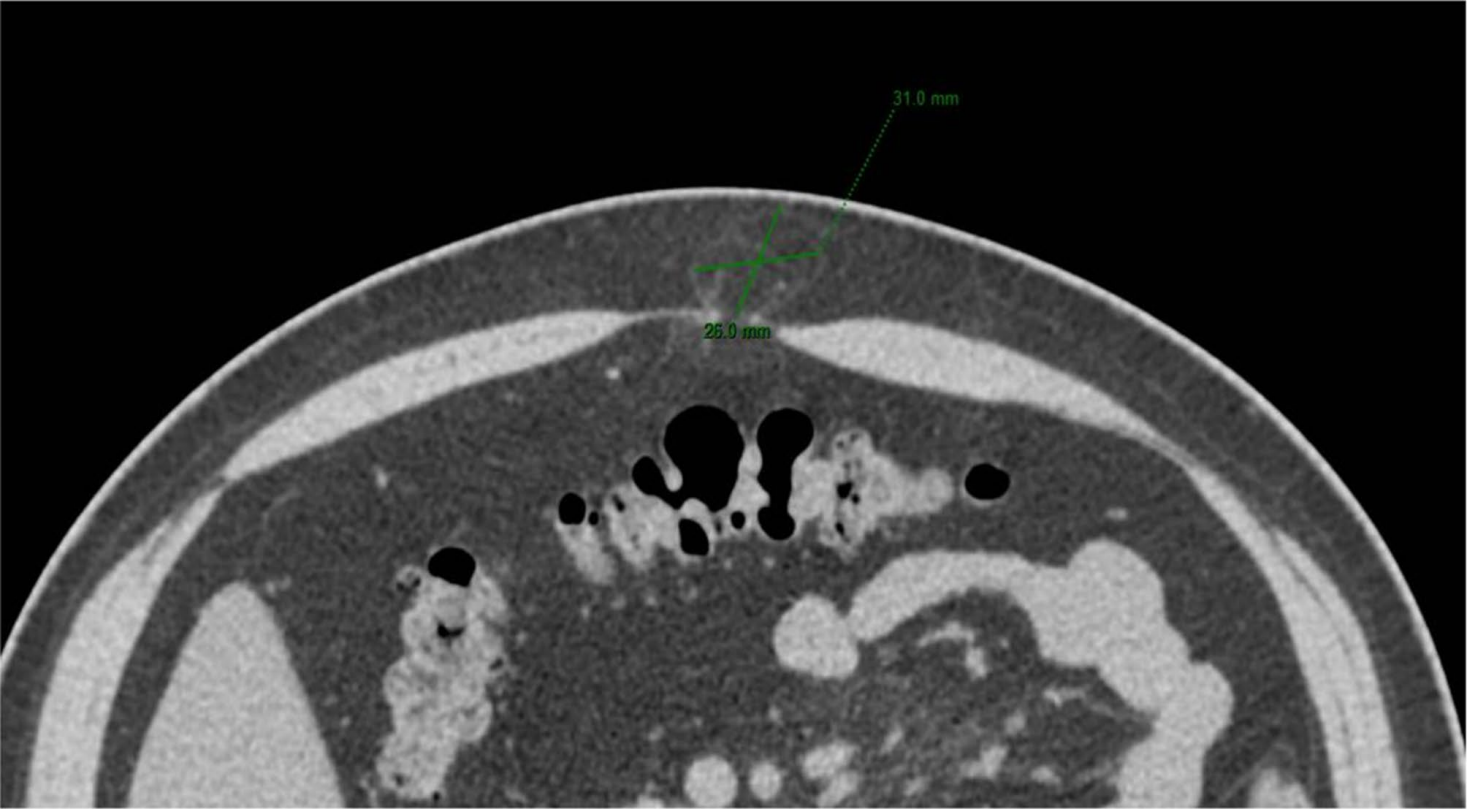
Preoperative computed tomography (CT).

Patient data were collected and reported according to the Strengthening the Reporting of Cohort Studies in Surgery (STROCSS) cohort study guidelines [19]. The diagnosis of an abdominal wall hernia was made based on symptoms, signs, and clinical examination. All patients received a preoperative abdominal computer tomography (CT) scan measure the size (width) of the defect (Fig. 1). Data extracted from the medical records included patient age, sex, body mass Index (BMI), history of alcohol use and smoking, hernia type (umbilical hernia, linea alba hernia, or incisional hernia), and comorbidities (diabetes and hypertension). Operation time and intraoperative blood loss data were extracted from the records. Postoperative data compared included analgesic dosage(tramadol), length of hospital stay, postoperative pain level as determined by a visual analog scale(VAS) pain score (a scale of 0 to 10; no pain to severe pain), complications, and hospitalization expenses. VAS pain score was assessed 12 and 24 h after surgery, and on postoperative day 7. All patients received an intravenous drip of 40 mg parecoxib postoperatively. Patients with a VAS pain score is greater than 5 or patients who ask for pain relief will take 50 mg tramadol additionally each time. The patient’s discharge criteria are the VAS score less than 4, and there has no special discomfort.

### Surgical procedures

Experienced surgeons with at least 10 years of experience in laparoscopic procedures and who performed more than 20 cases for IPOM plus or e-TEP procedures performed all of the surgeries. For both procedures were performed under general anesthesia with the patient in the supine position.

**Fig. 2:**
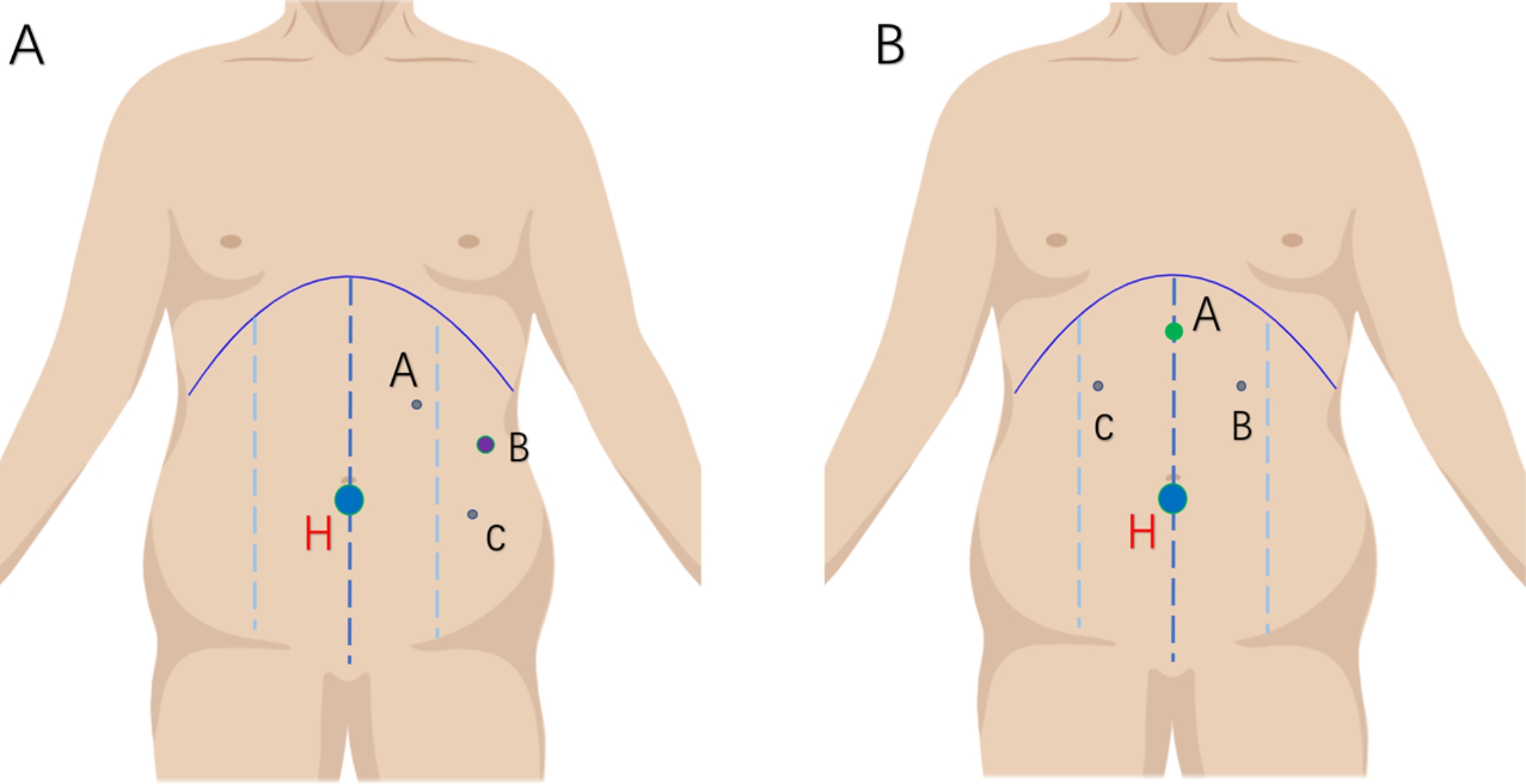
Port positions for e-TEP and IPOM plus. **A**: Port positions for e-TEP. H = hernia, B = 10 mm camera port, A and B are 5-mm working ports. **B**: Port locations for IPOM plus. H = hernia. A = the 10 mm camera port. B and C = 5-mm working ports

**Fig. 3:**
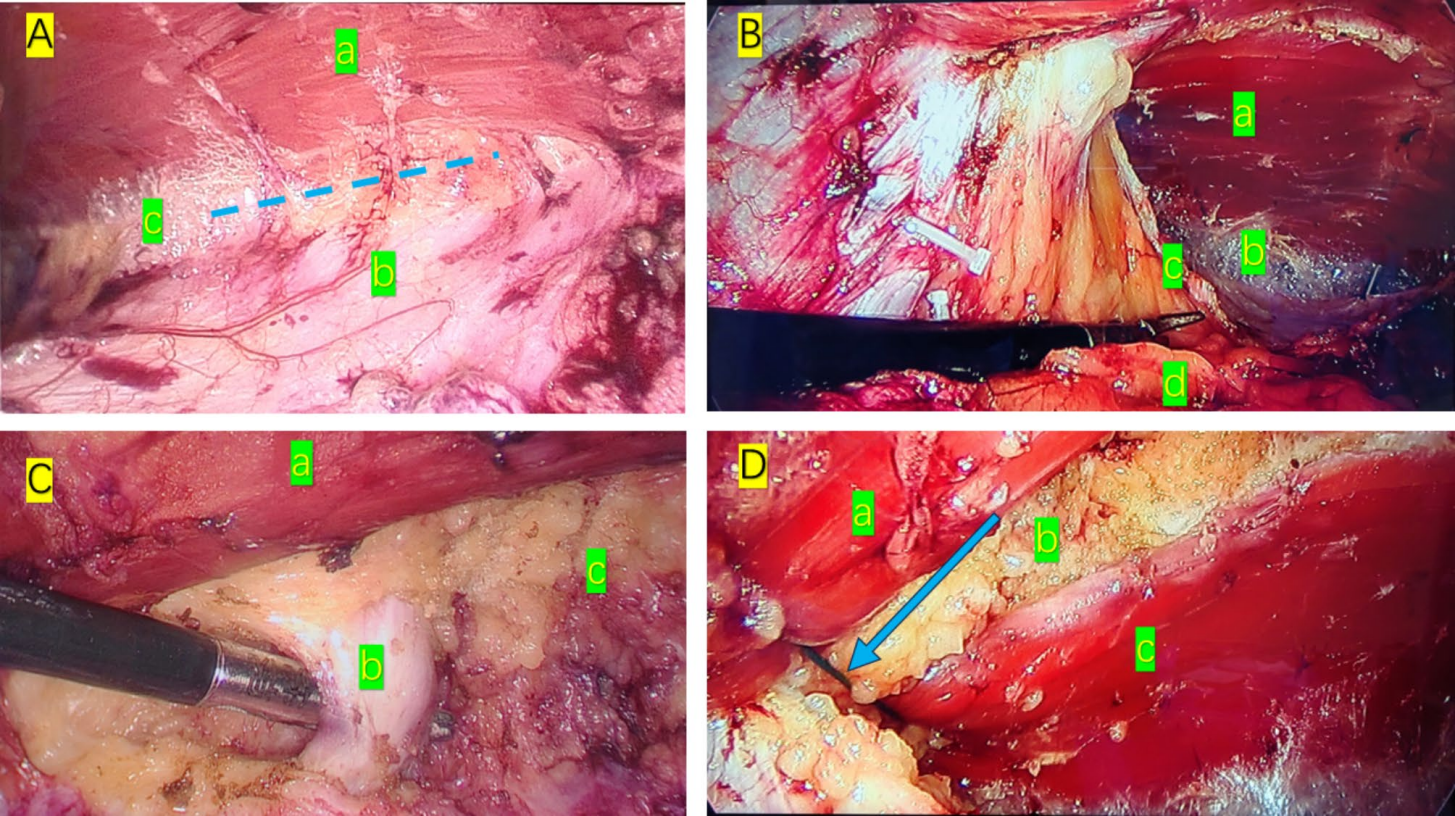
**A**: (a) Left rectus muscle. (b) Posterior sheath of rectus abdominis. c)Left retrorectus space (The dotted line is the direction of dissection). **B**: (a) Right rectus muscle (b) Right retrorectus space (c) Posterior sheath of the rectus muscle (d) Preperitoneal space. **C**: (a) Left rectus muscle (b) Umbilical hernia sac (c) Linea alba. **D**: (a) Left rectus muscle (b) Linea alba (c) Right rectus muscle (The arrow points to the non-absorbable suture that closes the abdominal wall defect)

For e-TEP, the choice of dissection area depended on the hernia’s location. For the hernias above the umbilical level, The right retrorectus space was used and dissected in the left lower quadrant for hernias around the navel or hypogastrium. In general, the initial incision was placed according to the location of the hernia defect. Take Infraumbilical defects as an example. Making an incision in the skin as the camera port medial to the anterior axillary line about 5 cm above the navel. After the incision of the skin and subcutaneous tissue, blunt dissection to the preperitoneal space, and a 10-mm trocar and a 30° laparoscope is inserted into the preperitoneal space under vision. Next, a CO2 pneumoperitoneum of 13 mm Hg is established while laparoscopic dissection is performed. After sufficient dissection, a 5-mm working port is placed about 8 cm lower than the camera port, and another 5-mm working port is placed slightly medial to the linea semilunaris, about 5 cm below the costal margins (Fig. 2. A). Both working ports are created under the laparoscopic vision to avoid damaging vital blood vessels. An incision is made on the medial side of the posterior rectus sheath, and dissection is performed toward the abdominal midline in the left retrorectus space (Fig. 3. A). Dissection is continued to cross over the linea alba at a depth of about 5 mm of the linea alba to expose the right posterior rectus sheath. An incision is made in the posterior sheath of the right rectus muscle, and the preperitoneal space around the hernia is enlarged with the extent of the dissection extending to both sides of the semilunar line (Fig. 3. B).

Reduction of the hernia sac is begun after obtaining sufficient room in the retrorectus space (Fig. 3C). If the hernia sac is tightly adherent, or the sac cannot be reduced for other reasons, an incision is made in proximal to the sac and the contents of the hernia sac are reduced into the abdominal cavity.

**Fig. 4:**
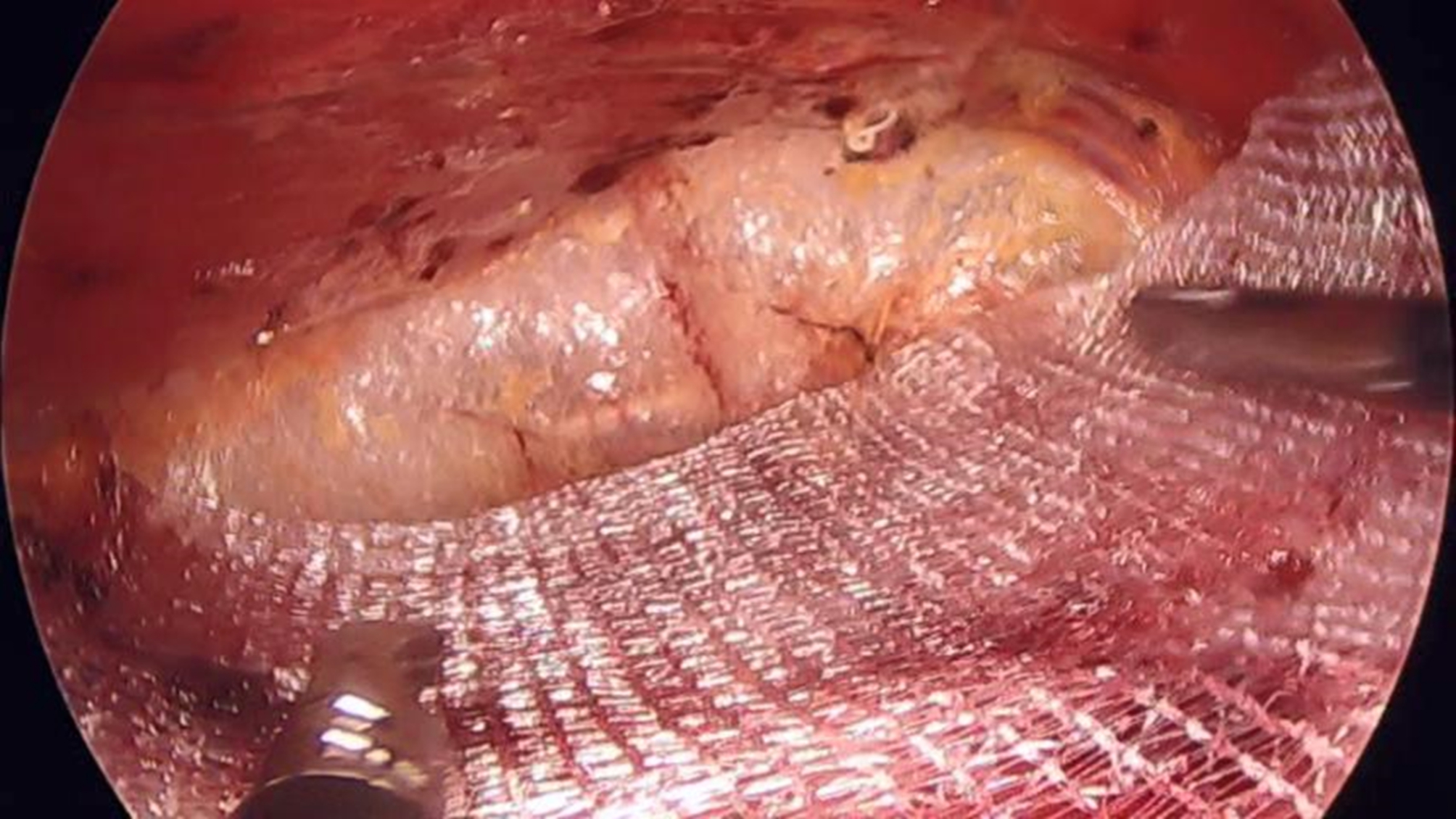
Mesh placement in the retrorectus space

After the contents are retracted, the abdominal wall defect is repaired. A disposable closure device (Covidien 173,022) using a non-absorbable suture (Johnson ETHIBOND X519H) is used to close the peritoneum of the abdominal wall defect (Fig. 3.D). Complete hemostasis is maintained throughout the procedure. A self-fixing polypropylene mesh (Covidien) measuring 15 × 9 cm is used to strengthen the abdominal wall. Lay the mesh on the defect in retrorectus space, with the edge of the mesh more than 5 cm from the defect edge in each direction. In general, no other fixation measures are needed to hold the mesh in place (Fig. 4). After laying the mesh, we routinely place a drainage tube in the anterior peritoneal space to prevent seroma or blood accumulation. The pneumoperitoneum is slowly released under laparoscopic vision to be certain the mesh remains in place, and then the incisions are closed with suture.

For the IPOM plus procedure, 3 incisions are made and a pneumoperitoneum of 13 mm Hg is established. The first 10 mm incision for the camera port is made about 5 cm below the xiphoid, and two 5 mm incisions are made bilaterally 3–5 cm below costal margins and in the midline of the clavicle as working ports (the 3 ports for a triangle; Fig. 2B). The hernia sac is identified and retracted into the abdominal cavity, and the defect is closed by the same method as described for e-TEP with a relatively low-pressure pneumoperitoneum (about 10 mm Hg). The abdominal wall is reinforced with a 15 × 10 cm polyester mesh with an anti-adhesive coating (Covidien). The mesh is then secured to the abdominal wall with an auto-suture fixation device (Covidien ProTack 174,006) after it is ensured that the mesh extends 5 cm past the hernia defect in all directions. After ensuring hemostasis, the pneumoperitoneum is released under the laparoscopic visualization, and the incisions are closed.

### Statistical analysis

Continuous data were presented a mean ± standard deviation, and the Kolmogorov-Smirnov test was used to determine the normality of distribution. Normally distributed data were compared with a t-test of calibrated t-test (if equal variances were not assumed). Non-normally distributed data were compared with the Mann–Whitney test. Categorical data were compared with the chi-squared test or Fisher’s exact test, as appropriate. Statistical analysis was performed using IBM SPSS version 27 software. A 2-tailed value of P < 0.05 was considered to indicate a statistically significant difference.

## Results

A total of 53 patients with ventral hernias were included: 22 patients underwent e-TEP, and the other 31 patients underwent IPOM plus. The mean age (48.1 ± 4.6 vs. 50.2 ± 4.9 years), and male: female ratio (13:9 vs. 19:12) of the 2groups were similar. The mean BMI in the IPOM plus group and e-TEP group was approximately 27 kg/${\text{m}}^{2}$. The distribution of hernia types (umbilical, linea alba, incisional) and frequency of comorbidities (diabetes, hypertension) was similar between the groups, as was the proportions of patients who used alcohol or smoked. The mean size of the hernia defect in the IPOM plus group was 4.1 cm, and in the e-TEP group was 3.9 cm, and the difference was not statistically significant. Patient characteristics are summarized in Table 1.

**Table 1:** Baseline characteristics of patients

Variables	(n = 22)	IPOM plus (n = 31)	*P-* value
Mean age (y)	48.1 ± 4.6	50.2 ± 4.9	0.116
Male: female	13:9	19:12	0.872
Mean BMI (kg/${m}^{2}$)	27.4 ± 1.9	27.0 ± 2.8	0.835
Umbilical: linea alba: incisional hernia	5:11:6	8:17:6	0.793
Mean defect size (width in cm)	3.9 ± 0.4	4.1 ± 0.6	0.213
Comorbidity (Diabetes: Hypertension)	7(D:H = 1:6)	12(D:H = 3:9)	0.910
Alcoholism(N)	7	11	0.781
Smoker(N)	6	10	0.697

n: number, y: years, e-TEP: extended view totally extraperitoneal repair, IPOM plus: intraperitoneal on lay mesh plus repair

**Fig. 5:**
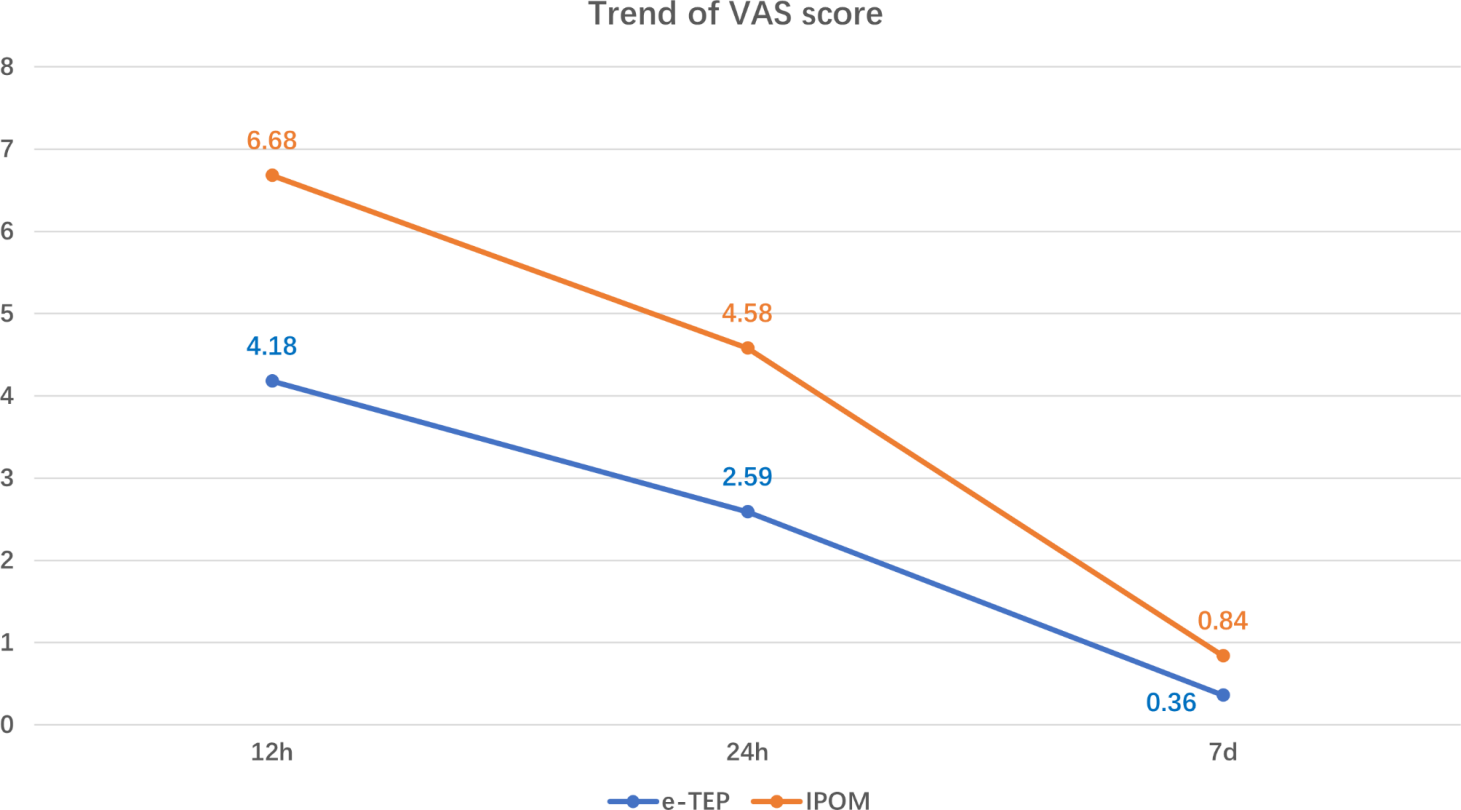
Visual analog scale (VAS) pain scores of the 2 groups at the 3 timepoints

The mean operation time in the IPOM plus group was significantly less than in the e-TEP group (65.9 ± 7.3 min vs. 98.5 ± 10.7 min, p < 0.05). The latter procedure takes more time than the former. The postoperative pain VAS scores at 12 h, 24 h, and 7 days after surgery indicated patients in the e-TEP group experienced less pain than those in the IPOM plus group (Fig. 5). Patients in the IPOM plus group used more tramadol than those in the e-TEP group (Tramadol: 72.6 ± 40.5 mg vs. 25.0 ± 37.0 mg). The mean length of hospital stay in the e-TEP group was significantly less than in the IPOM plus group 1.2 ± 0.5 vs. 2.2 ± 0.6 days). The total hospitalization cost was significantly less in the e-TEP group (19,695.9 ± 1221.8 vs. 35,286.2 ± 1196.5 CNY). Only one patient in the e-TEP group developed a seroma after surgery; no other postoperative complications were observed in either group in the first 7 days after surgery. Perioperative data are summarized in Table 2.

**Table 2:** Perioperative details

Variables	e-TEP (n = 22)	IPOM plus (n = 31)	*P*-value
Mean operative time (min)	98.5 ± 10.7	65.9 ± 7.3	< 0.05
Mean Blood loss (ml)	17.5 ± 7.2	18.2 ± 9.1	0.728
Mean VAS Score at:			
12 h after surgery	4.2 ± 0.9	6.7 ± 1.0	< 0.05
24 h after surgery	2.6 ± 0.9	4.6 ± 0.8	< 0.05
7 day after surgery	0.4 ± 0.5	0.8 ± 0.6	< 0.05
Dosage of Tramadol after surgery (mg)	25.0 ± 37.0	72.6 ± 40.5	< 0.05
Mean length of stay after surgery (days) Total hospital expenses (CNY) Postoperative complications	1.2 ± 0.5 19695.9 ± 1221.7 1 Subcutaneous fluid	2.2 ± 0.6 35286.2 ± 1196.5 -	< 0.05 < 0.05

n: number, VAS: Visual Analog Scale, CNY: Chinese yuan e-TEP: extended view totally extraperitoneal repair, IPOM plus: intraperitoneal on lay mesh plus repair

## Discussion

Since the in troduction of laparoscopic hernia repair, approaches for abdominal wall hernia repair with various techniques and different materials have rapidly developed [20]. The main components of the IPOM procedure are to reduce the content of the hernia sac and fix a composite mesh covering the defect intraperitoneally to cover the defect with non-absorbable tacks or sutures. The fascial closure technique with IPOM reinforcement is referred to as IPOM plus. The main difference between IPOM plus and IPOM is that with IPOM plus the abdominal wall defect is repaired using non-absorbable suture material before fixing the mesh [21]. It was found that IPOM plus was associated with a lower recurrence rate than IPOM [22]. As a result, the International Endohernia Society (IEHS) recommended IPOM plus for laparoscopic ventral hernia repairs, and included it in their 2014 guidelines [23].

Daes et al. [13] optimized the totally extraperitoneal (TEP) hernia repair technique, created the e-TEP technique for abdominal wall hernia repair, and obtained satisfactory results in 2012. Compared to TEP, the e-TEP procedure uses a different position for the camera port and cuts the Douglas’ line (arcuate line), which greatly increases the surgical field of view and makes the procedure less difficult [24]. Belyansky et al. [14] first applied the e-TEP technique to repair the ventral and incisional hernias, and confirmed its safety and reliability. The e-TEP technique allows the mesh to be placed in the retromuscular space, which prevents the mesh from contacting the abdominal cavity and thus avoids mesh-related complications. Also, due to where the mesh is placed, ordinary polypropylene meshes instead of composite meshes can be used, reducing the cost of the surgery [25].

Few clinical studies have compared e-TEP and IPOM for abdominal wall hernia repair; we found only 2 articles on the topic in the literature. Penchev et al. [26] studied 54 patients; half underwent e-TEP and half underwent IPOM. The postoperative pain scores were higher in the IPOM group, while the mean operation time was greater in the e-TEP group (186 vs. 90 min). There were no recurrences in the e-TEP group, and one recurrence in the IPOM group. With respect to postoperative complications, 4 patients in the e-TEP group and 3 in the IPOM group developed seromas after surgery. A study by Kumar et al. [16] reported similar results. In the Kumar study, 46 patients underwent e-TEP and 46 IPOM. The mean defect size in the e-TEP group was 3.9 cm and in the IPOM group was 4 cm, and the mean operation time in the e-TEP group was 107.52 min and in the IPOM group was 75.83 min. The postoperative VAS scores and length of hospital stay were both significantly less in the e-TEP group compared to the IPOM group. There were 6 patients who developed a seroma after surgery and 2 patients experienced a recurrence in the e-TEP group. In our study, postoperative pain scores, length of hospitalization, and hospital costs were significantly less in the e-TEP group; however, the operation time in the e-TEP group was significantly greater than in the IPOM group (95.8 ± 10.7 vs. 65.9 ± 7.3 min). It is likely that a drainage device was routinely placed after the operation, and the relatively short follow-up period resulted in only 1 patient in e-TEP group developed a postoperative seroma. Perhaps by increasing the sample size and follow-up time, the incidence of postoperative complications would be more accurate.

We included the total hospital costs as a research variable, and found the mean total hospital expenses for the IPOM plus group were significantly greater than for the e-TEP group. Because in the e-TEP procedure the mesh is placed outside of the peritoneal cavity, a composite mesh with anti-adhesive coating and a special fixing device is not required, making the total cost of e-TEP much less than IPOM plus.

Postoperative pain is a common complication of IPOM[27, 28]. In IPOM, the mesh needs to be fixed to the abdominal wall with staples, tacks, or transfascial sutures, and these fixation methods and mesh reactions are closely related to acute and chronic pain [15, 29]. Pain is relatively less after e-TEP because the mesh is not fixed and is not placed in the peritoneal cavity, and thus the foreign body reaction is relatively mild [12]. The studies by Penchev et al. and Kumar et al. also found low pain levels in patients who received the e-TEP procedure. In our study, pain scores were less in the e-TEP group at each timepoint they were measured. In addition, the IPOM plus group required more tramadol after surgery and had a longer length of hospital stay. Furthermore, only 3 ports are used in the e-TEP procedure, which likely results in less discomfort to a certain extent.

This study was a short-term observational study, and did not compare the differences in postoperative abdominal adhesions, however, some studies have examined this topic [30–33]. Placing a mesh in the abdominal wall defect can significantly reduce the recurrence rate of hernias and avoid wide fascial dissection and flap creation [31, 32]. However, if the mesh is placed inside the peritoneal cavity, there is a high probability of adhesiolysis-related complications and increased difficulty if a repeat surgical intervention is necessary [30]. Although many meshes with anti-adhesive coatings and special fixing materials are available, intraperitoneal adhesions can still occur after IPOM [29]. It is always necessary to consider the possibility of a repeat operation after intraperitoneal mesh placement. It is believed that e-TEP can overcome this problem because the mesh is placed in the preperitoneal space [17, 34]. The biggest limitation of e-TEP is that the operation time is longer. We found the mean time for the e-TEP procedure in our study was 98.5 ± 10.7 min, which is much longer than the IPOM operation time (65.9 ± 7.3 min). On the other hand, e-TEP is also more difficult than IPOM, requiring the operator to learn advanced laparoscopic skills and improve through continuous simulation exercises [33, 35].

Our findings showed that e-TEP is a viable and relatively more safe operation as compared to IPOM plus. A lower postoperative pain levels, shorter hospital stays, and lower hospitalization costs are the obvious advantages of e-TEP. The only disadvantage of e-TEP is the long operation time. Due to the short follow-up time of this study, many long-term complications after surgery, such as chronic pain and abdominal adhesions, were not examined; however, placing the mesh in an extraperitoneal location seems to reduce long-term complications. The single-center study, small number of patients, and short follow-up time are the limitations of this study. More multi-center, large-scale, long-term randomized control trials are required to find more beneficial evidence of e-TEP.

## Conclusions

As a new technique for ventral hernias repair, except for the longer operation time, e-TEP appears to be better than IPOM plus in terms of postoperative pain, dosage of postoperative analgesics length of hospital stay, and total cost.

## Electronic supplementary material

Below is the link to the electronic supplementary material.

Additional File 1: Ethics Statement

## Data Availability

The datasets used and analyzed for the current study are available from the corresponding author upon reasonable request.

## List of abbreviations

BMI: Body Mass Index
CNY: Chinese Yuan
e-TEP: Extended view totally extra peritoneal
IEHS: The International Endohernia Society
IPOM: Intraperitoneal on lay mesh
STROCSS: Strengthening the Reporting of Cohort Studies in Surgery
VAS: Visual analogue scale
