# Supplementary material for: Efficacy of extended view totally extra peritoneal approach versus laparoscopic intraperitoneal on lay mesh plus for abdominal wall hernias: a single center preliminary retrospective study
Source: BMC Surg. 2023 Jul 13;23:200. doi: 10.1186/s12893-023-02098-0 (PMC10339658; doi:10.1186/s12893-023-02098-0)
Supplement: Supplementary file 1 — Additional File 1: Ethics Statement [file 12893_2023_2098_MOESM1_ESM.pdf]

Ethics Statement

The study "Efficacy of extended view totally extra peritoneal approach versus laparoscopic intraperitoneal on lay mesh plus for abdominal wall hernias: a single center preliminary study" was approved by the Ethics Committee of The Second Medical College of Jinan University, Shenzhen People's Hospital. The Ethics Committee of The Second Medical College of Jinan University, Shenzhen People's Hospital specifically approved that written informed consent was required. Written informed consent for the surgical procedure and whether participated in Laparoscopic abdominal hernia repair group was signed by each patient or guardian, which was stored in the hospital database. The process was supervised by the Ethics Committee of The Second Medical College of Jinan University, Shenzhen People's Hospital.

Medical ethics committee number: LL-KW-20220102

Chairman of ethics committee:

2022-01-02
